# Anomaly detection of cybersecurity behavior using cross-sequence aligned transformer—A dynamic recognition approach for high-frequency interaction patterns

**DOI:** 10.1371/journal.pone.0340801

**Published:** 2026-02-18

**Authors:** Songming Han, Dongmei Bin, Ying Ling, Cong Lin

**Affiliations:** 1 Electric Power Research Institute of Guangxi Power Grid Co., Ltd., Nanning, Guangxi, China; 2 Guangxi Power Grid Co., Ltd., Nanning, Guangxi, China; Oakland University, UNITED STATES OF AMERICA

## Abstract

In high-frequency interaction network environments, network traffic features and user behavior sequences often exhibit pronounced temporal asynchrony and information redundancy, which can substantially weaken the capability of anomaly detection models to identify dynamic attack patterns. Based on this observation, this study proposes and empirically validates a core hypothesis: explicitly modeling the temporal asynchrony among multi-source sequences and performing collaborative modeling on a unified temporal scale can effectively enhance the accuracy and stability of cybersecurity anomaly detection under high-frequency interaction scenarios. To verify this hypothesis, a Cross-Sequence Aligned Transformer-driven Dynamic Recognition Model (CSAT-DRM) is developed, which falls within the category of deep learning–based multimodal time-series anomaly detection frameworks. The proposed model employs a cross-sequence alignment mechanism to softly align network traffic sequences and user behavior sequences, capturing their latent correlations without compressing inherent temporal discrepancies. Meanwhile, an interaction-sensitive residual structure is introduced into the Transformer encoding process to enhance the discriminability of anomalous features under high-frequency interactions, and a dynamic threshold generation strategy is integrated to enable adaptive anomaly discrimination. Experiments are conducted on real-world network interaction log data and evaluated through multiple baseline models and five independent repeated runs. The results show that CSAT-DRM achieves an accuracy of 0.968 ± 0.004, a precision of 0.957 ± 0.005, a recall of 0.953 ± 0.006, and an F1-score of 0.955 ± 0.005 on the test set, significantly outperforming baseline approaches including Long Short-Term Memory (LSTM), Convolutional Neural Networks (CNNs), the standard Transformer, and the hybrid Convolutional Neural Network–Bidirectional Long Short-Term Memory (CNN-BiLSTM) model. Further analysis demonstrates that the proposed model can effectively detect both burst anomalies and persistent anomalies, while maintaining high stability across different anomaly types. These findings validate the effectiveness of cross-sequence alignment and adaptive discrimination mechanisms in high-frequency interaction network anomaly detection, providing a feasible and generalizable technical pathway for real-time threat identification in complex network environments.

## 1. Introduction

The detection of anomalies in cybersecurity functions as a vital defensive measure for maintaining stable information system operations. The findings not only closely correlate with the safety degree of networked settings but also impact the upholding of core data completeness, consistent backing for infrastructure services, and uninterrupted progress in social and economic endeavors [1]. In the context of constantly enlarging global network size and more common high-frequency exchanges, irregular traffic and assault tactics show escalating intricacy and covert nature, which renders monitoring and protection efforts increasingly demanding. Established techniques based on predefined rules or simple statistical models often cannot adequately reconcile accuracy with speed when analyzing high-dimensional, diverse-source data possessing nonlinear traits [2,3]. Therefore, designing a effective, durable, and dynamically adjustable cybersecurity anomaly detection framework is necessary to further smart threat detection and formulate a scientifically sound protection strategy for information systems.

In deep learning–driven cybersecurity detection research, existing studies have attempted to combine deep feature extraction models with optimization or fusion strategies for the identification and classification of malware and network attack samples, thereby improving detection performance in complex security scenarios [4]. As a sequence modeling framework based on self-attention mechanisms, the Transformer has gradually emerged as a prominent approach in anomaly detection, owing to its strengths in global dependency modeling and multimodal feature fusion enabled by self-attention [5]. Certain research utilizes multi-head attention mechanisms to strengthen the representation of user behavior patterns, attaining higher accuracy compared to LSTM in detecting zero-day attacks and complex intrusions [6,7]. Other works combine Transformer with convolutional neural networks (CNNs), establishing layered interactions between local features and global dependencies to significantly boost the recall rate for internal threat identification [8,9]. Moreover, hybrid Transformer frameworks have been adopted for multimodal feature modeling, merging traffic logs, access behaviors, and contextual information into a unified representation space, which increases detection precision and lowers the risk of information loss [10,11].

However, existing Transformer-based anomaly detection methods typically rely on an implicit assumption during multimodal fusion that different feature sequences are temporally synchronized or can be directly aligned along the time dimension—an assumption that often fails to hold in high-frequency interaction network environments. Reconstruction-based Transformer models, exemplified by Transformer-based Anomaly Detection (TranAD), primarily focus on multivariate sequence modeling under a single or unified temporal scale and lack explicit mechanisms to handle the inherent temporal misalignment between traffic features and user behavior sequences. In contrast, approaches such as the Multimodal Spatial-Temporal Graph Attention Network (MST-GAT), which integrate graph attention with temporal modeling, are capable of capturing structural relationships across modalities; nevertheless, they still depend on fixed or implicit alignment strategies in temporally asynchronous scenarios. This reliance can easily lead to imbalanced attention allocation, thereby weakening the model’s ability to discriminate both burst anomalies and persistent anomalies. These limitations become particularly pronounced in high-frequency interaction scenarios, where they not only hinder the fine-grained characterization of anomalous patterns but also constrain the model’s adaptability and stability in dynamic environments.

To address the aforementioned limitations, this study develops a Cross-Sequence Aligned Transformer-driven Dynamic Recognition Model (CSAT-DRM) tailored for high-frequency interaction scenarios. By introducing a cross-sequence alignment mechanism, the proposed model enables collaborative modeling of network traffic sequences and user behavior sequences on a unified temporal scale without forcibly compressing inherent temporal discrepancies. In addition, the integration of an interaction-sensitive residual structure and a dynamic threshold–based discrimination strategy enhances the model’s adaptive responsiveness to high-frequency fluctuations and anomaly-inducing patterns, thereby enabling more sensitive and robust anomaly identification in complex network environments. The experimental findings validate the effectiveness of cross-sequence alignment mechanisms and adaptive discrimination strategies in cybersecurity anomaly detection, offering new insights and methodological support for the development of intelligent and highly adaptive cybersecurity defense systems.

This manuscript follows a structured organization: Section 1 outlines the study’s context, research questions, and aims; Section 2 comprehensively examines advancements in applying deep learning for detecting behavioral anomalies in cybersecurity; Section 3 details the dataset origins, development of the CSAT-DRM framework, and metric selection; Section 4 demonstrates experimental setups and outcome evaluations, particularly assessing CSAT-DRM’s effectiveness in identifying cyber threats during intensive interaction environments; Section 5 engages in critical discourse by comparing results with prior literature, highlighting both the study’s innovations and constraints; Section 6 summarizes key findings and suggests potential avenues for subsequent investigations.

## 2. Literature review

The detection of anomalies in cybersecurity is of central importance for ensuring the stable operation of information systems, guarding against internal threats, and recognizing complex attacks [12]. In this field, deep learning has become an essential research direction, owing to its superior performance in representing complex features, capturing long-term dependencies, and fusing heterogeneous multi-source data [13]. These developments have not only allowed for more profound analysis and dynamic detection of abnormal patterns but have also introduced new strategies to increase the timeliness and robustness of detection, highlighting considerable potential for further growth.

One of the key differentiating capabilities of deep learning lies in its aptitude for feature learning, which proves particularly valuable in analyzing complex, high-dimensional network traffic that lacks predefined structure. In the context of anomaly detection, the extraction of meaningful deep semantic features plays a vital role in boosting model performance. Lu et al. (2022) employed Variational Deviation Network (VDN) as an alternative to conventional reconstruction error methods, achieving more precise anomaly score learning [14]. Lin et al. (2022) focused on mitigating feature redundancy by developing horizontal and vertical dimension reduction approaches, which enhance both the effectiveness of feature representation and diagnostic performance [15]. Li et al. (2023) integrated CNN and ResNet architectures for semantic feature extraction, followed by Support Vector Machine (SVM) classification, enabling high-accuracy anomaly detection in unsupervised scenarios [16]. To enhance the distinction between normal and anomalous instances within latent space, Nguyen et al. (2024) designed a hierarchical nested clustering framework. This structure boosted the effectiveness of clustering-based detection systems [17]. Separately, Nkashama et al. (2024) introduced an autoencoder model with increased robustness, which directed the clustering of normal samples in latent representations. This approach improved resilience against data contamination and markedly lowered false positive rates [18].

In the field of cybersecurity, modeling temporal dynamics is critically important, particularly when detecting persistent and progressively evolving attacks. A detection system based on Continuous Temporal Graph Neural Networks (CTG) was developed by Duan et al. (2024). This framework effectively handles complex interaction patterns in 5G networks, where nodes frequently connect and disconnect, and achieves precise modeling of low-frequency activities [19]. Do et al. (2024) developed a deep model evaluation approach for dynamically evolving networks, maintaining model assessability while performing anomaly detection [20]. Rajendran et al. (2024) compared CNN and Recurrent Neural Network (RNN) architectures for detecting insider threats and zero-day attacks, finding that RNNs demonstrate superior performance when processing sequential behavioral data [21]. Khalaf et al. (2024) validated the effectiveness of hybrid CNN-RNN and autoencoder models using KDD Cup 1999 Intrusion Detection Dataset for time-window anomaly detection, achieving a 95% improvement in detection accuracy [22]. Wang et al. (2025) performed a thorough review of anomaly detection in multivariate time series. Their work analyzed deep learning-based temporal modeling methods, including architectures like LSTM, Transformer, and autoencoder variants. The survey highlighted the significance of temporal relationships within high-dimensional network data and identified key future needs, particularly in enhancing model generalization and explainability [23].

To tackle issues like heterogeneous multi-source data, imbalanced distributions, and varied attack patterns, a range of deep learning models with fusion and adaptive mechanisms have recently been developed for robust detection in complex attack environments. Yadav et al. (2023) introduced the AMAD approach, which conducts weighted CNN feature learning on fused data from visible-light, thermal, and infrared sources, showing superior results in low-quality settings [24]. Xi et al. (2023) captured implicit relationships using dynamic graph structures and performed joint modeling of correlated and uncorrelated features through dual autoencoders, notably enhancing detection accuracy [25]. Momynkulov et al. (2024) created a hybrid multi-architecture detection framework that integrates CNN, RNN, and attention mechanisms to accomplish feature extraction, threat localization, and automated mitigation across multiple data channels, effectively lowering false alarm rates [26]. Ahmed et al. (2024) integrated an adaptive feature aggregation module into their DELM model and used generative networks to handle class imbalance, greatly improving malicious traffic detection [27]. Liu et al. (2024) suggested an adaptive feature fusion mechanism for high-dimensional variant data in power systems, successfully reducing modeling complexity and speeding up anomaly identification [28]. Beyond end-to-end deep models, some studies have explored performance improvement pathways from the perspective of model optimization and detector co-design. Alzubi et al. (2023) introduced Harris Hawks Optimization into malware detection tasks, jointly optimizing feature weights and classifier parameters to enhance discriminative capability [29]. Subsequently, Movassagh et al. (2023) further combined deep feature extraction networks with optimization strategies to achieve effective detection and classification of network attacks and malicious samples. This line of research primarily focuses on sample-level discriminative performance and parameter optimization, providing a valuable complement for improving detection accuracy [30].

Current investigations in attribute learning, time-series modeling, and dynamic fusion techniques have provided essential theoretical and procedural support for this study, particularly in behavioral pattern analysis, dependency modeling, and heterogeneous feature combination. Nevertheless, in cybersecurity environments with rapid interactions, conventional research predominantly employs fixed-threshold approaches, which are inadequate in coping with the dynamic variations of complex network data flows. Consequently, detection systems often exhibit limited real-time responsiveness and precision. To mitigate these shortcomings, this research prioritizes cross-sequence feature harmonization and self-adjusting discrimination mechanisms, establishing a deep learning detection model optimized for high-frequency interaction contexts. The objective is to achieve more accurate and resilient anomaly identification in multifaceted network ecosystems.

## 3. Research design

### 3.1. Data source and preprocessing

The data used in this study were obtained from a network interaction log repository jointly established by a large-scale Internet enterprise and a collaborating security laboratory, covering six consecutive months of operational data from January 2024 to June 2024. The raw data were stored in the form of unstructured logs, containing both network traffic information and user behavior records. The study first performed unified parsing and structural transformation of the raw logs, mapping textual records into standardized fields, while discarding invalid samples with severe missing fields or abnormal formatting.

During the data cleaning stage, mean imputation was applied to repair a small proportion of randomly missing numerical features. For extreme outliers that deviated substantially from the overall distribution, trimming based on the three-sigma rule was employed to reduce the adverse impact of outlier values on training stability. These cleaning rules were applied consistently across the entire dataset, without manual adjustments for specific categories or time periods.

To mitigate the temporal asynchrony between network traffic sequences and user behavior sequences under high-frequency interaction scenarios, a sliding time window of 5 seconds was uniformly adopted for event aggregation and resampling, ensuring alignment of interaction information from different sources on a unified temporal scale. Subsequently, all input features were subjected to standardization and normalization to eliminate scale differences and maintain numerical distribution stability, thereby providing a consistent input space for subsequent model training.

After completing log parsing, data cleaning, temporal alignment, and feature normalization, approximately 45 million valid samples were obtained, including around 35 million normal behavior samples and 10 million potentially anomalous or suspicious samples. To prevent data leakage and ensure objective evaluation, the dataset was split into training, validation, and test sets according to a 7:2:1 ratio, with all subsets maintaining consistent temporal distributions and class proportions.

### 3.2. Model construction

**Fig 1:**
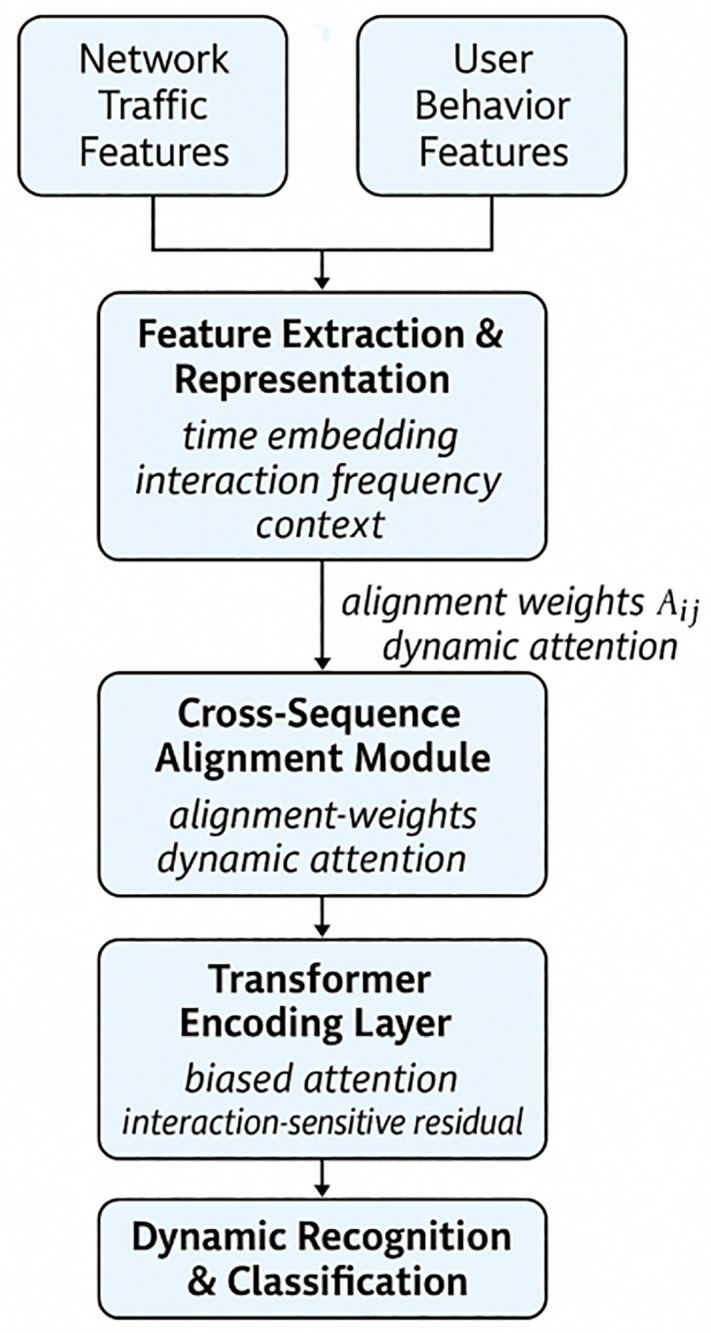
CSAT-DRM Framework Diagram.

This paper presents the Cross-sequence Aligned Transformer-driven Dynamic Recognition Model (CSAT-DRM) for high-frequency interaction scenarios. At the feature extraction and representation stage, the model first integrates timestamps, interaction frequency, and contextual data to achieve multi-dimensional representation, projecting them uniformly into a shared modeling space. Following this, a cross-sequence alignment module leverages dynamic attention to address asynchronous relationships between traffic and behavior sequences and to accentuate potential anomaly patterns. The Transformer encoding layer further utilizes cross-sequence biased attention and interaction-aware residual mechanisms to model global dependencies and preserve robustness under frequent interactions. Finally, a dynamic recognition and classification layer enables sensitive detection of anomalous behaviors via anomaly scoring, flexible thresholds, and decision rules, establishing an integrated detection system that combines accuracy with applicability. The CSAT-DRM framework is shown in Fig 1.

#### 3.2.1. Feature Extraction and Representation.

In anomaly detection tasks for network security behavior, the quality of features fundamentally influences model effectiveness. Due to the marked asynchrony and diversity between network traffic and user behavior in high-frequency interaction environments, this research designs a three-stage feature extraction and representation procedure: raw feature extraction → temporal and interaction pattern encoding → sequence representation mapping. This approach ensures that the input representations retain temporal relationships while supplying ample information for later cross-sequence alignment and Transformer-based modeling. The detailed steps are outlined below:

1. Raw Feature Extraction

For network behavior data, this paper extracts features from two dimensions: traffic features and user behavior features. The traffic features include packet size *p*_*t*_, protocol type *proto*_*t*_, session duration *d*_*t*_, and packet interval time *Δt*_*t*_; while the behavior features include user operation type *a*_*t*_, resource access pattern *r*_*t*_, and sensitive port or resource *flag*_*t*_.

The raw features at time step t can be represented as:


$$x_{t}=[p_{t},\Delta t_{t},proto_{t},d_{t},a_{t},r_{t},flag_{t}]$$ (1)

where *t* denotes the time-step index, which is used to characterize the relative ordering of events within the sliding time window.

The complete input sequence is:


$$X=\{x_{1},x_{2},\ldots ,x_{T}\},\;x_{t}\in R^{d}$$ (2)

where *T* denotes the total number of behavioral events within the time window, and *d* represents the dimensionality of the raw feature vector at each time step.

2. Temporal and Interaction Pattern Encoding

To capture the temporal irregularity and sporadic nature of malicious activities, this study introduces two supplementary feature representations: time-aware embeddings and frequency-based interaction encodings:

1. Timestamp Embedding

A periodic function mapping approach is adopted to enable the model to recognize both periodic and aperiodic burst features:


$$TE(t_{i})={\left[\sin\left(\frac{t_{i}}{\tau^{2k/d_{te}}}\right),\cos\left(\frac{t_{i}}{\tau^{2k/d_{te}}}\right)\right]}_{k=1}^{d_{te}/2}$$ (3)

where *t*_*i*_ denotes the event timestamp, τ represents the time scale factor, *k* denotes the embedding dimension index, and *d*_*te*_ represents the total dimensionality of the temporal embedding. The operator ${\left[\;\cdot \;\right]}_{k}^{\frac{d_{te}}{\text{2}}}$ indicates the concatenation of sine and cosine components at different frequencies along the embedding dimension, forming the temporal embedding vector.

2. Interaction Frequency Embedding

To reflect behavioral density under high-frequency interaction patterns, the interaction frequency per unit time is defined as:


$$IF(i)=\log\left(1+\frac{N_{i}}{\Delta t_{i}+\varepsilon }\right)$$ (4)

where *N*_*i*_ indicates the number of interactions within a time period, *Δt*_*i*_ represents the interaction interval, ε serves as a smoothing term to prevent zero denominators, log(⋅) denotes the natural logarithm operation.

3. Context-Enhanced Representation

To further characterize anomalous behavior patterns in cybersecurity scenarios, contextual information embedding is introduced:


$$Ctx(i)=[flag_{i},port_{i},risk_{i}]$$ (5)

where *flag*_*i*_ denotes the sensitive operation marker, *port*_*i*_ represents the accessed port category, and *risk*_*i*_ indicates the rule-based risk weight.

4. Unified Embedding Representation

The raw features, temporal encoding, interaction frequency, and contextual information are concatenated:


$${\hat{x}}_{i}=[x_{i}⊕TE(t_{i})⊕IF(i)⊕Ctx(i)]$$ (6)

where ⊕ denotes the vector concatenation operation.

Then, linear transformation is applied to map it to the Transformer’s modeling dimension:


$$z_{i}=W_{e}{\hat{x}}_{i}+b_{e},\;z_{i}\in R^{d_{model}}$$ (7)

where *W*_*e*_ and *b*_*e*_ denote the learnable projection weight matrix and bias term, respectively, and *d*_*mode*l_ represents the hidden representation dimensionality of the Transformer.

yielding the complete input sequence:


$$Z=\{z_{1},z_{2},\ldots ,z_{T}\}$$ (8)

#### 3.2.2. Cross-Sequence Alignment Module.

Under rapid interaction conditions, behavioral logs typically contain network traffic subsequences and user activity subsequences, which frequently display time offsets and redundant information. Raw input to Transformer models may cause attention bias, obscuring latent anomalous signals. Our solution is the Cross-Sequence Alignment Module (CSA), which implements a dynamic weighting strategy to harmonize traffic and behavior features temporally. The alignment process involves:

1. Sequence Partition

Let the input sequence obtained through the aforementioned feature extraction and representation be denoted as $Z=\{z_{1},z_{2},\ldots ,z_{T}\}$. Based on its source, it can be divided into:

Traffic feature subsequence: $Z^{\left(f\right)}=\{z_{1}^{\left(f\right)},z_{2}^{\left(f\right)},\ldots ,z_{m}^{\left(f\right)}\}$

Behavioral feature subsequence: $Z^{\left(a\right)}=\{z_{1}^{\left(a\right)},z_{2}^{\left(a\right)},\ldots ,z_{n}^{\left(a\right)}\}$

where *m* and *n* denote the lengths of the traffic feature subsequence and the behavioral feature subsequence, respectively, and typically satisfy *m* ≠ *n*, reflecting the temporal asynchrony in the sampling processes of the two sequences.

2. Dynamic Alignment Weight Calculation

To resolve the temporal misalignment problem, we propose an alignment attention matrix. A bidirectional correlation mechanism evaluates the interaction strength between the two subsequences across varying time intervals:


$$A_{ij}=\frac{\exp\left(\langle z_{i}^{\left(f\right)},z_{j}^{\left(a\right)}\rangle /\sqrt{d_{model}}\right)}{\sum_{k=1}^{n}\exp\left(\langle z_{i}^{\left(f\right)},z_{k}^{\left(a\right)}\rangle /\sqrt{d_{model}}\right)}$$ (9)

where *Aij* denotes the alignment weight between the *i*-th traffic feature step and the *j*-th behavioral feature step. This alignment mechanism does not forcibly compress events from different modalities into the same timestamp; rather, it constitutes an attention-based soft alignment process. During the computation of alignment weights, the model retains the original timestamps and interaction density features, allowing the natural temporal ordering and anomaly-induced asynchronous shifts to be learnably encoded into the aligned representations. In this way, the approach avoids weakening genuine temporal discrepancies under a shared temporal representation. The inner product ⟨⋅,⋅⟩ is used to measure feature similarity.

3. Cross-Sequence Alignment Representation

After obtaining the alignment weight matrix A, the traffic sequence can be mapped to the semantic space of the behavioral sequence, thereby forming the aligned representation:


$${\tilde{z}}_{i}^{\left(f\right)}=\sum_{j=1}^{n}A_{ij}\cdot z_{j}^{\left(a\right)}$$ (10)

Similarly, the behavioral sequence can be aligned to the traffic sequence:


$${\tilde{z}}_{j}^{\left(a\right)}=\sum_{i=1}^{m}A_{ij}\cdot z_{i}^{\left(f\right)}$$ (11)

where ${\tilde{z}}_{i}^{\left(f\right)}$ and ${\tilde{z}}_{j}^{\left(a\right)}$ denote the traffic feature representation and the behavioral feature representation, respectively, after cross-sequence alignmen

4. Fusion and Anomaly Sensitivity Enhancement

To enhance sensitivity for anomaly detection tasks, this paper introduces an anomaly enhancement factor γ on the aligned representations, designed to amplify anomalous patterns in high-frequency interactions:


$$z_{i}^{\left(c\right)}=\gamma \cdot {\tilde{z}}_{i}^{\left(f\right)}+(1-\gamma )\cdot z_{i}^{\left(f\right)}$$ (12)

where *γ*∈(0,1) denotes the anomaly enhancement coefficient, which is used to balance the contributions of the aligned representations and the original representations.

The value of γ is dynamically adjusted based on interaction frequency embedding *IF*(i):


γ=σ(α·IF(i)) (13)

where *IF*(*i*) denotes the interaction frequency embedding at the i-th time step;

*σ* represents the Sigmoid function, and *α* is a learnable parameter used to regulate the influence of interaction frequency on the strength of anomaly enhancement.

#### 3.2.3. Transformer Encoding Layer.

After accomplishing cross-sequence alignment, the obtained aligned representation sequence $\hat{Z}=\{z_{1}^{\left(c\right)},z_{2}^{\left(c\right)},\ldots ,z_{T}^{\left(c\right)}\}$ requires further modeling of global dependencies to capture complex interaction patterns. The Transformer encoding layer introduced in this study incorporates cross-sequence attention bias and an interaction-aware residual mechanism, building upon the alignment outcomes. This design enhances both sensitivity and robustness for identifying anomalies in high-speed interaction environments. The implementation involves the following steps:

1. Cross-Sequence Biased Attention

During the attention computation, a Bias Cross-Sequence Attention matrix (BCSA) generated from the cross-sequence alignment module is introduced to strengthen cross-sequence dependencies between traffic and behavioral features:


$$H_{i}=Softmax\left(\frac{QK^{⊤}}{\sqrt{d_{k}}}+B_{CSA}\right)V$$ (14)

where Q, K, and V denote the Query, Key, and Value matrices, respectively. The values of BCSA are derived from the aforementioned alignment weight matrix *Aij*, ensuring that the encoding layer fully exploits cross-sequence information when modeling long-range dependencies. *Hi* represents the contextual representation obtained at the i-th time step through biased attention computation.

2. Interaction-Sensitive Residual Mechanis

To counteract the degradation of anomaly feature representations in residual pathways during rapid interactions, we develop an interaction-aware residual mechanism:


$$X_{i}^{\prime }=LayerNorm(X_{i}+\eta_{i}\cdot H_{i})$$ (15)

where *Xi* denotes the input feature before the residual connection, and LayerNorm(⋅) represents the layer normalization operation. The scaling factor *ηi* depends on the interaction frequency feature, defined as follows:


$$\eta_{i}=1+\beta \cdot IF(i)$$ (16)

where β is a learnable parameter. This mechanism enables the model to automatically amplify the weight of anomalous patterns in residual signals when interaction frequency is high.

3. Dynamic Feedforward Enhancement

An interaction-sensitive scaling factor is introduced into the feedforward network to ensure model stability during traffic burst scenarios:


$$FFN(h_{i})=\delta (W_{2}\cdot \sigma (W_{1}h_{i}+b_{1})+b_{2})\cdot (1+\lambda \cdot IF(i))$$ (17)

where *hi* denotes the input feature to the feedforward network; *W1* and *W2*, along with *b1* and *b2*, represent the weight matrices and bias terms of the feedforward network, respectively; and σ(⋅) denotes the nonlinear activation function. λ serves as an adjustment coefficient to moderately amplify the output of the feedforward layer under high-frequency interactions.

#### 3.2.4. Dynamic detection and classification layer.

After cross-sequence alignment and deep encoding, the resulting sequence representation $V=\{v_{1},v_{2},\ldots ,v_{T}\}$ comprehensively reflects the interaction patterns of traffic and behavioral features. However, in real-world cybersecurity scenarios, anomalies often manifest as dynamic patterns evolving over time and can be easily masked in high-frequency interactions. Therefore, this paper proposes a Dynamic Detection and Classification Layer, which employs a three-step process—anomaly scoring, dynamic threshold adjustment, and classification decision—to ensure robust and sensitive detection results. The detailed steps are as follows:

1. Anomaly Scoring Function

First, an anomaly scoring function is defined for each time step i:


$$S_{i}=f(v_{i})\cdot \left(1+\lambda \cdot IF(i)\right)$$ (18)

where *vi* denotes the feature representation output by the Transformer encoding layer at time step *i*. *f*(*vi*)denotes the anomaly probability output based on a multilayer perceptron (MLP); *IF*(*i*) represents the interaction frequency feature; and λ is a learnable scaling coefficient.

2. Dynamic Threshold Generation

To mitigate false positives or false negatives caused by fixed thresholds, this paper proposes a dynamic threshold mechanism based on interaction fluctuations:


$$\theta_{i}=\theta_{0}\cdot \left(1+\mu \cdot Var(IF[1:i])\right)$$ (19)

where θ₀ is the baseline threshold, Var(IF[1:i]) denotes the variance of interaction frequencies up to the current timestep, and μ is a tuning parameter.

3. Classification Decision

Based on the anomaly score and dynamic threshold, the final decision rule is defined as:


$$y_{i}=\{\;\;\;\begin{array}{cc} {}*20l1, & S_{i}\geq \theta_{i}\\ 0, & S_{i}<\theta_{i} \end{array}$$ (20)

where yᵢ = 1 indicates that the current timestep is classified as anomalous, while yᵢ = 0 denotes normal behavior.

### 3.3 Evaluation metrics

To thoroughly assess the effectiveness of the Cross-Sequence Aligned Transformer-Based Dynamic Recognition Model (CSAT-DRM), this research adopts a multi-dimensional evaluation framework. Key metrics encompass global accuracy, anomaly identification reliability, detection scope, and stability, as elaborated in the following sections:

1. Accuracy (ACC)

Classification Accuracy quantifies the model’s total prediction correctness by calculating the ratio of accurate predictions to all evaluated cases. As a fundamental and universally adopted benchmark, it provides a clear overview of the system’s effectiveness. The calculation is defined by:


$$Accuracy=\frac{TP+TN}{TP+TN+FP+FN}$$ (21)

where TP denotes the number of correctly identified anomalous samples, TN represents correctly classified normal samples, FP is the count of normal samples misclassified as anomalies, and FN is the number of anomalies mistakenly labeled as normal.

2. Precision (P)

Detection Precision measures the fraction of true anomalies within all instances flagged as anomalous, highlighting the model’s capacity to suppress false positives. In security applications, robust precision reduces operational overhead by avoiding erroneous alerts. The metric is computed as:


$$Precision=\frac{TP}{TP+FP}$$ (22)

3. Recall

Detection Sensitivity calculates the ratio of true anomalies correctly identified, demonstrating the model’s ability to prevent missed detections. In security systems, poor sensitivity leads to critical threats evading analysis, weakening overall protection. The computation follows:


$$Recall=\frac{TP}{TP+FN}$$ (23)

4. F1-score

F1-score is the harmonic mean of precision and recall, providing a balanced assessment of the model’s performance in handling false positives and false negatives. For imbalanced data distributions, F1-score offers a more reliable evaluation. The calculation is:


$$F1=\frac{2\times Precision\times Recall}{Precision+Recall}$$ (24)

## 4. Results and analysis

### 4.1. Experimental setup

The testing environment ran on Ubuntu 20.04 LTS, utilizing an Intel Xeon Gold 6226R processor (2.9 GHz), 256 GB RAM, and an NVIDIA Tesla V100 GPU (32 GB VRAM). The software stack consisted of Python 3.9 and PyTorch 1.12. Model weights were initialized with random values and refined through the Adam optimizer, configured with a starting learning rate of 0.0001, a batch size of 256, and a 50-epoch training limit. To enhance generalization, learning rate scheduling and early termination mechanisms were applied. The above parameter settings are consistent with the training configurations commonly adopted in recent Transformer-based time-series anomaly detection studies [5,31], and exhibit stable convergence behavior on the validation set. To guarantee the reliability of the findings, each test was conducted 5 separate times with the same configuration. The outcomes display the mean and standard deviation of all performance indicators, demonstrating the algorithm’s consistency across varying random weight initializations.

In terms of operational mode, CSAT-DRM adopts an online inference mechanism based on fixed-length sliding time windows, where all module computations are confined to a single time window and do not involve repeated backtracking over the entire historical sequence. Both the cross-sequence alignment and Transformer encoding processes are executed at the window level, with computational overhead growing linearly with the window length. The inference latency is therefore dominated by the time-window aggregation process rather than by additional blocking introduced by the model architecture itself. Under the aforementioned hardware configuration, the proposed model is able to meet the real-time anomaly detection requirements of high-frequency interaction scenarios.

During the simulation stage, the experiments are conducted under the following reasonable assumptions. First, it is assumed that under the specified high-performance hardware environment, the convergence behavior and performance of the training process are reproducible, and are not subject to significant variation across different runtime platforms. Second, it is assumed that after standardization and sliding-window resampling, traffic sequences and behavioral sequences maintain semantic consistency on a unified temporal scale, thereby supporting effective modeling via the cross-sequence alignment mechanism. Third, interaction frequency is assumed to faithfully reflect activity intensity and potential anomaly trends under high-frequency behaviors, such that the dynamic threshold generation can achieve adaptive performance with the support of this feature.

To ensure clear reproducibility of the experimental pipeline, all hyperparameters are fixed prior to final evaluation and remain consistent across all experiments and baseline models. All coefficient parameters introduced in the model formulations are treated as learnable parameters and are jointly optimized with network weights during training. Model selection and early stopping are conducted exclusively based on validation set performance, and no test set information is used during training or hyperparameter tuning, thereby avoiding potential data leakage and ensuring the objectivity and reproducibility of the experimental results.

### 4.2. Model performance analysis

#### 4.2.1. Convergence analysis.

**Fig 2:**
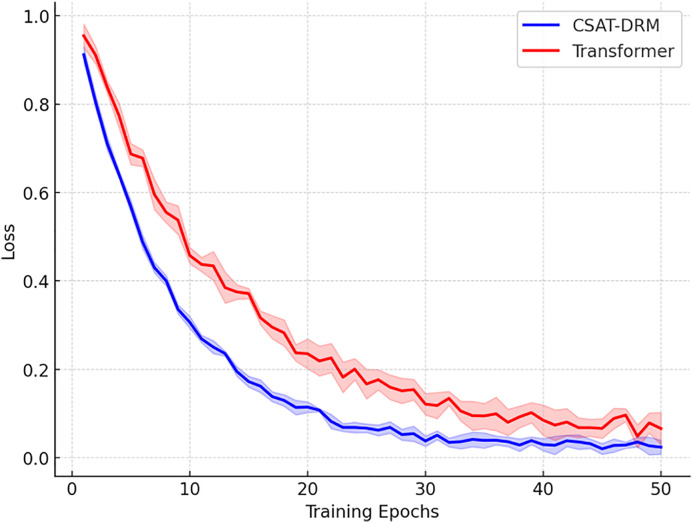
Convergence curve.

This research analyzes the training dynamics of the Cross-Sequence Aligned Transformer-Driven Dynamic Recognition Model (CSAT-DRM) against a conventional Transformer architecture, using the same experimental setup to assess convergence efficiency. As illustrated in Fig 2, CSAT-DRM achieves notably quicker early-stage convergence than the baseline Transformer. Its loss value drops sharply within the initial 10 training steps and plateaus by the 20th epoch, reflecting superior optimization stability. Conversely, the standard Transformer attains steady loss values only near the 30th epoch, with residual oscillations persisting in later phases. By integrating averaged metrics and deviation bounds from five trial runs, CSAT-DRM shows reduced variance across random initializations. Its convergence trajectory is markedly smoother, confirming that the model excels not only in accelerated training for high-frequency tasks but also in consistency and generalization strength relative to the benchmark.

#### 4.2.2. Model performance comparison.

In this study, LSTM, CNN, Transformer, and CNN-BiLSTM are selected as baseline models to evaluate the overall performance advantages of CSAT-DRM in cybersecurity anomaly detection tasks. LSTM serves as a representative recurrent neural network that can efficiently learn temporal dependencies in interaction behavior sequences, rendering it the most conventional baseline model for anomaly detection. CNN extracts features within localized windows using convolutional kernels, featuring parameter sharing and high computational efficiency—this demonstrates how traditional deep models perform in spotting local anomalous patterns. Transformer, a extensively adopted sequence modeling framework in recent years, captures global dependencies through self-attention, displaying strong expressive capacity in tasks involving network behavior analysis and constituting the most straightforward reference for evaluating the utility of our proposed modifications. As a widely adopted hybrid deep structure in recent years, CNN-BiLSTM combines the strengths of CNNs in local feature enhancement with those of BiLSTM in bidirectional temporal dependency modeling, enabling a more comprehensive representation of hybrid modeling capabilities in high-frequency interaction scenarios. The inclusion of CNN-BiLSTM therefore facilitates a more rigorous assessment of the performance gains achieved by CSAT-DRM in cross-scale behavioral pattern representation.

**Fig 3:**
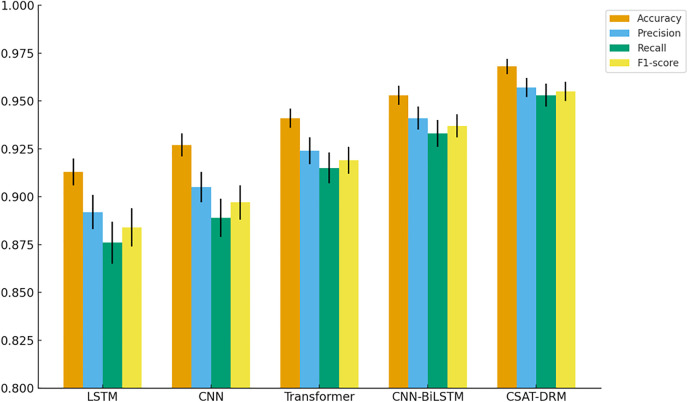
Comparative results of model performance.

As illustrated in Fig 3, CSAT-DRM achieves the best performance across all four evaluation metrics and significantly outperforms the baseline models, including LSTM, CNN, Transformer, and the hybrid Convolutional Neural Network–Bidirectional Long Short-Term Memory (CNN-BiLSTM) architecture. In terms of Accuracy, CSAT-DRM attains 0.968 ± 0.004, representing improvements of approximately 5.5%, 4.1%, and 2.7% over LSTM, CNN, and Transformer, respectively. Even when compared with the relatively strong CNN-BiLSTM model (0.953 ± 0.005), CSAT-DRM still achieves an improvement of about 1.5%. This advantage is primarily attributed to the introduction of the cross-sequence alignment mechanism, which enables the model to integrate contextual dependencies between traffic sequences and behavioral sequences on a unified temporal scale, thereby reducing misclassifications caused by temporal misalignment and semantic information loss in conventional models.

With respect to Precision, CSAT-DRM reaches 0.957 ± 0.005, markedly outperforming the baseline models (with CNN-BiLSTM achieving the highest precision among them at 0.941 ± 0.006), yielding an overall improvement of approximately 1.6%–6.5%. This performance gain mainly stems from the interaction-sensitive residual structure, which automatically amplifies high-frequency interaction features with anomalous tendencies. As a result, clearer inter-class boundaries are formed in the high-dimensional feature space, effectively reducing false positives. In contrast, CNN-based models are prone to overfitting local window features, while recurrent models often introduce redundant information during long-sequence modeling, both of which may increase false alarm rates.

Regarding Recall, CSAT-DRM achieves 0.953 ± 0.006, corresponding to improvements of 7.7%, 6.4%, and 3.8% over LSTM, CNN, and Transformer, respectively, and still about 2.0% higher than CNN-BiLSTM (0.933 ± 0.007). These results indicate that under scenarios characterized by sparse anomaly samples and complex anomaly feature distributions, the proposed model is able to capture a larger proportion of latent anomalous patterns, thereby effectively reducing missed detections. The synergistic effect of cross-sequence feature alignment and global attention mechanisms plays a key role in driving this improvement.

In terms of the F1-score, CSAT-DRM attains 0.955 ± 0.005, achieving improvements of approximately 3%–7% over the baseline models and demonstrating superior overall detection capability. As a balanced metric integrating precision and recall, the F1-score further confirms that CSAT-DRM can maintain stable overall performance while simultaneously suppressing false alarms and missed detections. The combined effects of cross-sequence semantic consistency modeling and anomaly-sensitive feature enhancement mechanisms enable the model to consistently converge to near-optimal solutions across multiple independent experimental runs.

### 4.3. Ablation study

To assess the individual contributions of key components, we performed an ablation study, with outcomes presented in Table 1. The complete CSAT-DRM model attained the highest scores across all four evaluation metrics, reaching an accuracy of 0.968 ± 0.004 and an F1-score of 0.955 ± 0.005, while also showing the lowest standard deviation. These findings indicate that the model not only delivers high detection accuracy but also maintains consistent robustness across repeated trials. In contrast, omitting the cross-sequence alignment module caused a general decline of 2–4% in both accuracy and recall, with recall falling to 0.917 ± 0.009. This suggests that without alignment, the model cannot adequately utilize temporal correlations across multimodal features, leading to the omission of certain anomalous instances. When the interaction-sensitive residual module was excluded, precision dropped to 0.931 ± 0.008 and the F1-score decreased to 0.928 ± 0.007, reflecting a reduction of 2–3 percentage points relative to the full model. This confirms that the residual structure plays a vital role in sharpening feature discriminability and lowering false positives, where normal instances are incorrectly flagged as anomalies. Additional tests using single-modal inputs further validated the importance of multimodal fusion: using only traffic features or only behavioral features reduced overall performance by 4–6% compared to the complete model. Specifically, the traffic-only version achieved an accuracy of 0.925 ± 0.007, while the behavior-only variant performed even lower at 0.918 ± 0.008. This implies that single-modal data alone cannot adequately represent complex interaction patterns, and that cross-modal fusion is necessary to integrate complementary information and substantially boost overall model effectiveness.

**Table 1:** Ablation experiment results.

Model	Accuracy	Precision	Recall	F1-score
Full model	0.968 ± 0.004	0.957 ± 0.005	0.953 ± 0.006	0.955 ± 0.005
w/o Cross-Sequence Alignment	0.942 ± 0.006	0.928 ± 0.007	0.917 ± 0.009	0.922 ± 0.008
w/o Interaction-Sensitive Residuals	0.947 ± 0.005	0.931 ± 0.008	0.926 ± 0.007	0.928 ± 0.007
Traffic only	0.925 ± 0.007	0.902 ± 0.009	0.894 ± 0.010	0.898 ± 0.009
Behavior only	0.918 ± 0.008	0.896 ± 0.010	0.887 ± 0.011	0.891 ± 0.010

### 4.4. Analysis of anomaly detection results

**Fig 4:**
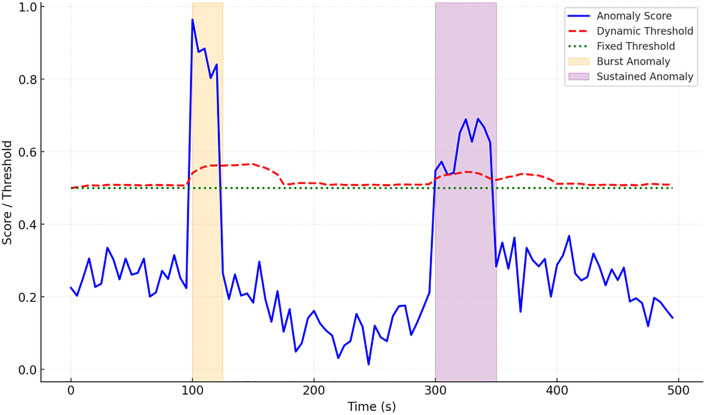
Dynamic Performance of Anomaly Detection in Time-series.

We carried out time‑series dynamic performance tests to further evaluate the model’s responsiveness in continuous traffic scenarios, focusing especially on the temporal evolution of anomaly scores and detection thresholds. Fig 4 displays the results: the anomaly score curve (blue) peaks abruptly between t = 20–25 s and exhibits a prolonged increase from t = 60–70 s, matching burst anomalies and persistent anomalies, respectively. The dynamic threshold (red dashed line) adapts in real time to score fluctuations—it quickly elevates during burst anomalies to mitigate false positives triggered by short‑term traffic spikes, and gently declines as interaction frequency drops to preserve detection capability for subtle potential anomalies. Conversely, a static threshold (green dotted line) remains unchanged regardless of traffic variations over time, making it susceptible to erroneous judgments in periods of sparse anomalies or localized fluctuations. Further statistical evaluation across repeated runs reveals that the dynamic threshold not only sustains a consistent score‑threshold equilibrium across different time windows, but also facilitates near‑instant identification of newly emerging anomaly signals, markedly boosting real‑time detection performance and dependability. The results confirm that the model exhibits considerable adaptability and robustness along the temporal dimension, successfully trading off sensitivity and stability in intricate network environments. This capacity provides a solid basis for follow‑up anomaly classification and defensive measures, underscoring the model’s practical feasibility for cybersecurity applications.

**Fig 5:**
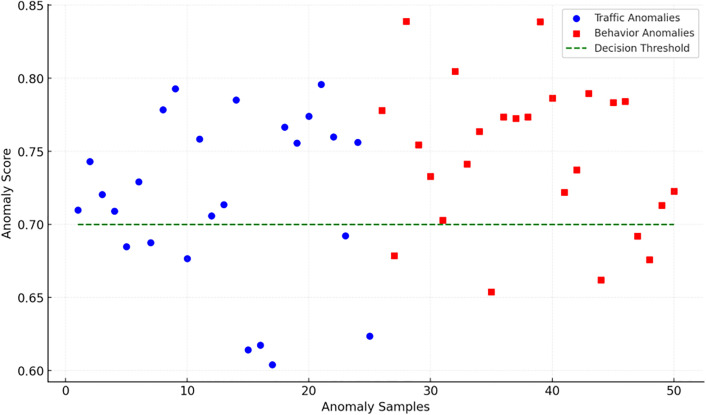
Detection score distribution across different anomaly types.

Additional testing supported the model’s competence in differentiating between two anomaly types. As presented in Fig 5, scores for both kinds of anomalies stayed above the threshold throughout, proving the model’s effectiveness in spotting anomalous occurrences. Distribution patterns differed, however: behavior-level anomalies exhibited higher scores with clearer divergence trends, whereas traffic-level anomalies partially coincided near the threshold boundary. These results show that through multimodal fusion, CSAT-DRM can jointly capture abrupt traffic changes and behavioral pattern deviations, enabling the simultaneous identification of dissimilar anomaly types. By employing cross-sequence alignment, the model boosts the synergistic relationship between traffic and behavioral characteristics in a shared temporal context, upholding high distinguishability for both anomaly categories within the decision space.

### 4.5. Computational overhead and runtime efficiency analysis

To evaluate the engineering feasibility of CSAT-DRM in practical cybersecurity monitoring systems, an additional analysis of the model’s computational overhead is conducted from three perspectives: training-stage computational cost, resource consumption, and online inference efficiency. The corresponding results are summarized in Table 2. During the training stage, the computational overhead of CSAT-DRM is primarily concentrated in the cross-sequence alignment module and the attention computations and intermediate feature buffering within the Transformer encoding layers. The overall training process converges within several hours, and since training is performed offline and does not participate in the online detection pipeline, it does not impose any impact on the real-time performance of the deployed system.

**Table 2:** Computational Cost and Runtime Efficiency of CSAT-DRM.

Category	Metric	Value	Description
Training Cost	Training time per epoch	15–20 min/ epoch	Batch size = 256, including forward and backward propagation
Training Cost	Total training time	8–12 h	With early stopping, approximately 40–50 epochs
Resource Usage	Peak GPU memory (training)	22–26 GB	Approximately 70%–80% of V100 (32 GB)
Resource Usage	GPU memory (inference)	6–8 GB	Forward inference and window-level buffering only
Resource Usage	Peak CPU memory	35–45 GB	Including data loading and feature caching
Runtime Efficiency	Inference latency per window	40–60 ms/ window	Sliding window length = 5 s
Runtime Efficiency	Inference throughput	≈ 15–25 windows/s	Single-GPU setting
Model Size	Number of parameters	≈ 18–20 M	Including cross-sequence alignment and Transformer encoder
Reproducibility	Independent runs	5	Same setting as main experiments

With respect to resource consumption, GPU memory usage reaches its peak during training due to the need to maintain gradient information and intermediate feature representations. In contrast, during the inference stage, only network weights and window-level intermediate representations required for forward computation are retained, resulting in substantially reduced GPU memory and system memory requirements. Under deployment environments with relatively sufficient resources—such as servers or security operation centers—the model can operate stably without imposing excessive pressure on system resources.

In terms of runtime efficiency, CSAT-DRM adopts an online inference mechanism based on fixed-length sliding time windows, where all computations are confined to a single window and no backtracking over the full historical sequence is involved. Cross-sequence alignment, attention computation, and anomaly scoring are all executed at the window level, with computational complexity increasing linearly with the window length. Under these conditions, the model is able to complete inference within a single window, thereby satisfying the real-time anomaly detection requirements of high-frequency interaction scenarios.

### 4.6. Model robustness under cross-dataset conditions

To further examine the stability and generalization capability of the proposed model under different data distribution conditions, additional comparative experiments are conducted on the public benchmark intrusion detection dataset CIC-IDS2017. Since this dataset does not provide complete user behavior logs, the experiments are performed using network traffic–related features only to construct time-series inputs, while preserving the overall architecture and dynamic discrimination mechanisms of CSAT-DRM. The evaluation metrics and baseline model settings are kept consistent with the previous experiments. All experiments are independently repeated five times under identical conditions, and the mean values and standard deviations are reported.

As shown in Table 3, under the public dataset setting with traffic features only, CSAT-DRM consistently maintains performance advantages across Accuracy, Precision, Recall, and F1-score, while exhibiting relatively small standard deviations. This indicates strong result consistency and robustness under different random initialization conditions. Despite the absence of explicit user behavior sequence inputs, CSAT-DRM is still able to effectively characterize the evolution of anomalous patterns in traffic sequences through its cross-temporal alignment and dynamic threshold discrimination mechanisms. These results suggest that the performance improvements achieved by CSAT-DRM are not dependent on a specific enterprise environment or private log structure, but instead demonstrate a certain degree of generalization capability and stability across heterogeneous data distribut

**Table 3:** Performance comparison of different models on the CIC-IDS2017.

Model	Accuracy	Precision	Recall	F1-score
CNN	0.931 ± 0.006	0.918 ± 0.007	0.905 ± 0.008	0.911 ± 0.007
LSTM	0.938 ± 0.005	0.924 ± 0.006	0.912 ± 0.007	0.918 ± 0.006
Transformer	0.944 ± 0.004	0.931 ± 0.005	0.919 ± 0.006	0.925 ± 0.005
CNN-BiLSTM	0.947 ± 0.004	0.935 ± 0.005	0.923 ± 0.006	0.929 ± 0.005
CSAT-DRM	0.952 ± 0.004	0.940 ± 0.005	0.928 ± 0.006	0.934 ± 0.005

## 5. Discussion

In high-frequency interaction environments, the presented Cross-Sequence Alignment Transformer-driven Dynamic Recognition Model (CSAT-DRM) successfully resolves the temporal misalignment between network traffic and user behavior sequences. Through the incorporation of interaction-sensitive residual connections and an adaptive threshold mechanism, the model markedly improves both the precision and resilience of anomaly identification. Empirical evaluations confirm that CSAT-DRM exceeds baseline models in comprehensive performance, with its superiority largely stemming from the combined effect of cross-sequence alignment and interaction-aware design. These contributions effectively mitigate challenges related to temporal desynchronization and feature overlap in high-frequency settings, resulting in enhanced stability and adaptability in dynamic detection scenarios and anomaly differentiation.

In comparison with existing literature, this investigation shows distinctive methodological and outcome-based contributions. Tuli et al. (2022) introduced TranAD, a Transformer-based model that advances multivariate time-series anomaly detection and highlights training efficiency [31]; however, it does not sufficiently account for temporal asynchrony in high-frequency interactions. Our cross-sequence alignment technique synchronizes traffic and behavioral features on a unified time scale, resulting in more accurate and responsive detection. Ding et al. (2022) proposed MST-GAT, which applies graph attention and temporal convolution to capture multimodal sequence relationships and elevates detection performance [32]. Yet, its reliance on fixed thresholding increases false alarm risks in burst-traffic scenarios. Our method resolves this by introducing a dynamic threshold generation scheme that adaptively modifies detection limits, significantly improving the false-positive–false-negative equilibrium. Yu’s DTAAD model (2024) achieves lightweight detection by coupling Transformer with dual TCNs [33], but remains sensitive to feature redundancy in high-dimensional contexts. By integrating an interaction-aware residual design that dynamically recalibrates channel weights according to interaction frequency, our model reduces interference from redundant features and preserves stable discriminative power. The comparative results of the key mechanism characteristics of the aforementioned models are presented in Table 4.

**Table 4:** Key mechanism comparison of transformer-based anomaly detection models.

Model	Core Mechanism	Cross-Sequence Alignment	Dynamic Threshold	Multi-Modal Fusion	Anomaly Type Detection
TranAD	Transformer + Reconstruction	—	—	Single-modal	Sudden anomaly
MST-GAT	Transformer + Graph Attention + TCN	—	—	Yes	Multiple anomaly types
DTAAD	Dual TCN + Attention Networks	—	—	Single-modal	Gradual anomaly
CSAT-DRM	Cross-Sequence Alignment Transformer + Interaction-Sensitive Residuals + Dynamic Threshold	Yes	Yes	Yes	Sudden, gradual, and hybrid anomalies

The key theoretical advancement of this research is the introduction of a cross-sequence alignment strategy, offering innovative perspectives for joint multimodal feature learning. This approach facilitates seamless fusion of network traffic data and user activity patterns within a shared temporal architecture, enhancing the interpretation of abnormal signatures in high-velocity interaction contexts. Additionally, the frequency-aware residual module emphasizes how interaction rates influence feature separability, pushing the traditional use of skip connections into new frontiers for anomaly identification. Furthermore, the adaptive thresholding system overcomes the constraints of static threshold methods, uncovering novel solutions for real-time, environment-responsive detection in dynamic time-series systems. From a practical application perspective, CSAT-DRM is not designed to replace human security analysts, but rather to function as an intelligent decision-support tool for continuous monitoring, anomaly screening, and risk prioritization of massive network behavior data in high-frequency interaction scenarios. The anomaly scores and dynamic threshold outputs generated by the model can provide security analysts in Security Operations Centers (SOCs) with rapid references, helping them focus on high-risk events and improve analysis efficiency, while the final threat assessment and response decisions still require integration of human expertise, business context, and security policies. Through this human–machine collaborative operational paradigm, CSAT-DRM can significantly alleviate the cognitive and temporal burden on analysts in high-frequency interaction environments, without compromising the flexibility and judgment inherent in human decision-making.

Although the proposed Cross-Sequence Alignment Transformer-driven Dynamic Recognition Model demonstrates superior performance across multiple experiments, certain limitations remain. First, the data used for model evaluation are primarily sourced from a single enterprise network and a laboratory-constructed log repository, where traffic patterns are dominated by office operations and research-and-development interactions. Network environments with markedly different characteristics—such as financial transactions, cloud platform access, industrial control systems, smart campuses, or Internet of Things (IoT) terminals—are not yet covered. While the architectural design of CSAT-DRM does not rely on industry-specific protocols or business logic and is therefore theoretically capable of cross-domain transfer, variations across industries in protocol types, user behavior patterns, interaction frequencies, traffic structures, and attack vectors may affect the effectiveness of the dynamic threshold adjustment mechanism and the interaction-sensitive residual modeling, potentially leading to performance discrepancies. Consequently, the stability and generalization capability of the model in cross-industry and multi-traffic-pattern environments still require systematic validation. Second, the incorporation of cross-sequence alignment and interaction-sensitive residual structures inevitably introduces additional computational overhead and a certain degree of dependence on hardware resources, which may constrain model deployment in resource-limited edge or terminal environments. From a computational perspective, this overhead is primarily concentrated on feature alignment and attention computation within a single sliding time window, without involving full-sequence backtracking across multiple windows. As such, the model is more suitable for deployment in online monitoring scenarios with relatively sufficient resources, such as servers or security operation centers. Finally, the present approach to feature modeling is limited to traffic and behavior, failing to include additional contextual elements—for example, geographical data, device attributes, or environmental factors. This omission partially hinders the model’s ability to generalize across diverse multimodal detection settings.

Future research directions could explore the following aspects. First, to address the current limitation that the model has only been validated in a single enterprise network environment, future work will incorporate real-world traffic data from domains such as financial systems, industrial control networks, cloud platforms, and the Internet of Things (IoT), which exhibit diverse protocol structures and behavioral patterns. This will enable a systematic evaluation of the model’s robustness and transferability across multi-industry scenarios. In addition, domain adaptation and multi-source transfer learning techniques will be explored to enhance the model’s ability to adapt to scenario heterogeneity, thereby improving cross-environment detection consistency and stability. Second, to reduce the model’s considerable computational cost, future work should explore more streamlined network designs or optimization techniques—such as model pruning and knowledge distillation—to lower hardware requirements and boost practical utility in resource-limited settings. Finally, concerning feature representation, gradually integrating broader contextual data—like device environment, user location, or security policy elements—through multimodal fusion and hierarchical modeling could widen the model’s perceptive scope and strengthen anomaly detection in intricate scenarios.

## 6. Conclusion

This study addresses the challenges of temporal asynchrony between network traffic and user behavior sequences and complex interaction relationships in high-frequency interaction network environments by developing a Transformer-based dynamic anomaly recognition approach that enables unified modeling and collaborative analysis of multi-source time-series information. The research primarily demonstrates that, under high-frequency interaction scenarios, explicitly characterizing the temporal relationships among heterogeneous sequences can effectively enhance the stability and discriminative consistency of anomaly detection in dynamic environments. Experimental results indicate that the proposed method maintains stable and competitive detection performance across diverse anomaly patterns and temporal evolution conditions. It is capable of simultaneously handling both burst anomalies and persistent anomalies, reflecting strong temporal adaptability and overall robustness. In complex interaction scenarios, unified modeling of multi-sequence temporal relationships helps alleviate the performance fluctuations commonly observed in traditional methods due to temporal misalignment and insufficient feature coupling. Overall, this study provides a feasible modeling paradigm for anomaly detection in high-frequency interaction network scenarios and validates the effectiveness of Transformer-based temporal collaborative modeling for analyzing complex.

## Supporting information

S1 Dataset Minimal dataset for CSAT-DRM training. (XLSX)
